# Analysis of copy number variants detected by sequencing in spontaneous abortion

**DOI:** 10.1186/s13039-024-00683-3

**Published:** 2024-05-20

**Authors:** Anhui Liu, Liyuan Zhou, Yazhou Huang, Dan Peng

**Affiliations:** 1 Hengyang Medical School, University of South China, Hengyang, 421000 China; 2 Department of Medical Genetics, Xiangya School of Medicine, Changde Hospital, Central South University (The First People’s Hospital of Changde city), Changde, 415000 China; 3 Hunan Provincial Key Laboratory of Regional Hereditary Birth Defects Prevention and Control, Changsha Hospital for Maternal & Child Health Care Affiliated to Hunan Normal University, Changsha, 410000 China

**Keywords:** Spontaneous abortion, Copy number variations, Candidate genes, *LZTR1*

## Abstract

**Background:**

The incidence of spontaneous abortion (SA), which affects approximately 15–20% of pregnancies, is the most common complication of early pregnancy. Pathogenic copy number variations (CNVs) are recognized as potential genetic causes of SA. However, CNVs of variants of uncertain significance (VOUS) have been identified in products of conceptions (POCs), and their correlation with SA remains uncertain.

**Results:**

Of 189 spontaneous abortion cases, trisomy 16 was the most common numerical chromosome abnormality, followed by monosomy X. CNVs most often occurred on chromosomes 4 and 8. Gene Ontology and signaling pathway analysis revealed significant enrichment of genes related to nervous system development, transmembrane transport, cell adhesion, and structural components of chromatin. Furthermore, genes within the VOUS CNVs were screened by integrating human placental expression profiles, PhyloP scores, and Residual Variance Intolerance Score (RVIS) percentiles to identify potential candidate genes associated with spontaneous abortion. Fourteen potential candidate genes (*LZTR1*, *TSHZ1*, *AMIGO2*, *H1-4*, *H2BC4*, *H2AC7*, *H3C8*, *H4C3*, *H3C6*, P*HKG2*, *PRR14*, *RNF40*, *SRCAP, ZNF629*) were identified. Variations in *LZTR1*, *TSHZ1*, and *H4C3* may contribute to embryonic lethality.

**Conclusions:**

CNV sequencing (CNV-seq) analysis is an effective technique for detecting chromosomal abnormalities in POCs and identifying potential candidate genes for SA.

**Supplementary Information:**

The online version contains supplementary material available at 10.1186/s13039-024-00683-3.

## Background

Spontaneous abortion is one of the most common complications of pregnancy, occurring in approximately 15% of pregnancies, defined as pregnancy loss before 28 weeks of gestation without human intervention [1, 2]. The etiology of SA is complex, involving genetic factors, autoimmune diseases, endocrine disorders, thrombophilias, and environmental factors [3–5]. Embryo chromosomal abnormalities, including numerical and structural chromosome abnormalities, as well as pathogenic copy number variations (pCNVs), play a primary role in early SA(< 12 weeks of gestation) [6, 7]. Numerical chromosome abnormalities are the most prevalent type of chromosome abnormalities [8], with pCNVs following closely [9]. CNVs which are the increase or decrease of DNA fragments larger than 1 kb bases on a chromosome, mainly in the form of deletions and duplications at the submicroscopic level are recognized as significant genetic variations strongly associated with the risk of SA [10–14]. In recent years, an increasing number of studies have demonstrated the association of CNVs with various complex and common disorders [10], such as neurodevelopmental disorders, autism, cancer, and Parkinson’s disease, by altering gene function [15–19].

Chromosome karyotype analysis, a fundamental test for identifying chromosome abnormalities as the underlying cause of malformations or diseases, has been utilized in POC samples for years. However, it has encountered increasing limitations such as low resolution, long cell culture cycles, and difficulty in detecting pathogenic microdeletions and microduplications smaller than 5 Mb [20]. The emergence of copy number variation sequencing based on next-generation sequencing (NGS) technology has addressed the shortcomings of traditional genetic detection methods, significantly improving detection efficiency and reducing misdiagnosis rates. Although pathogenic CNVs are recognized as causes of SA, the presence of numerous CNVs of uncertain significance detected in POCs remains to be explored in clinical trials [21, 22].

This study aims to systematically investigate the frequency and distribution differences of chromosomal abnormalities in SA and to explore the role of CNVs with unknown clinical significance. CNV-seq was employed to detect POC samples, and gene functions of both pathogenic CNVs and CNVs of uncertain significance were analyzed through enrichment and signaling pathway analyses. Genes within the VOUS region were further examined alongside gene conservation scores (PhyloP), tissue-specific gene expression, RVIS scores, and percentiles. The objective is to identify candidate genes associated with embryonic development or abortion and offer meaningful molecular genetic guidance for high-risk pregnancies.

## Materials and methods

### Participants

A total of 189 POC samples were collected from pregnant women experiencing spontaneous abortions, who were admitted to the First People’s Hospital of Changde City between January 2020 and November 2022. Informed consent was obtained from all participants, and the study was approved by the Medical Ethics Committee of the First People’s Hospital of Changde City. POC samples inclusion criteria (1) all patients with unexplained spontaneous abortion within 28 weeks of gestation, (2) no history of smoking or alcohol consumption, and (3) no history of taking teratogenic drugs and no history of exposure to toxic substances in the first three months of pregnancy or during pregnancy. POC samples exclusion criteria (1) significant maternal cell contamination, (2) coagulation disorders, endocrine abnormalities, and immune function abnormalities prior to pregnancy, (3) anatomical and structural malformations of the reproductive tract, (4) history of infectious diseases during pregnancy.

### CNV sequencing

Genomic DNA from peripheral blood cells was extracted using the DNeasy Blood & Tissue Kit (Qiagen) following the manufacturer’s instructions. The DNA sample concentration is greater than 8ng/μl (Qubit assay) and the total amount is not less than 50ng. A sequencing library was prepared using 50 ng of genomic DNA as a template. Initially, DNA was fragmented to an average size of 300 bp, followed by ligation of a 9 bp barcode sequencing adapter. Modified fragments underwent PCR amplification, and fragments were then selected and purified using bead purification to remove interference from primer dimers. Subsequently, a DNA library was constructed and the purified DNA library concentration should not be less than 25nM. CNV-seq was performed on the NextSeq CN500 platform (Berry Genomics). Sequences were mapped to the GRCh37 reference genome, which was conducted by the Burrows-Wheeler Alignment tool. Reads were processed and CNVs were evaluated by an in-house pipeline using read counts based on a smoothness model (Berry Genomics, Beijing, China) [23]. Copy number gains or losses were compared with in-house database of copy number variants (CNVs) and with public CNV databases, including Genomic Variants (http://dgv.tcag.ca/dgv/app/home), UCSC(https://genome.ucsc.edu/cgi-bin/hgGateway), NCBI(https://www.ncbi.nlm.nih.gov/), Decipher(http://decipher.sanger.ac.uk/),Online Mendelian Inheritance in.

Man (OMIM, http://www.omim.org/) and ClinGen (https://www.clinicalgenome.org/) [24, 25]. CNV segments with microdeletions or microduplications greater than 100 kb were recorded. All genomic coordinates were based on the Human GRCh37/hg19 Genome Assembly. The American College of Medical Genetics and Genomics (ACMG 2019) standard was utilized as the final criterion for evaluating the pathogenicity of CNVs. Finally, the distribution map of pCNVs and VOUS CNVs on chromosomes was generated using R version 4.02 software.

### Statistical analysis

Data analysis was conducted using SPSS software (version 29.0, IBM Corp., Armonk, NY, USA). Descriptive statistical methods were employed to present the data, with measurement data expressed as mean ± SD. A significance level of *P* < 0.05 was considered statistically significant.

### Functional enrichment analysis

Protein-coding genes within pathogenic CNVs, likely pathogenic CNVs, and VOUS regions were referenced from the DECIPHER (http://decipher.sa-nger.ac.uk/) and Clingen (http://www.ncbi.nlm.nih.gov/projects/dbvar/clingen/) databases. Gene ontology (GO) analysis and Kyoto Encyclopedia of Genes and Genomes (KEGG) analysis were conducted using the DAVID bioinformatics database (https://david.ncifcrf.gov). The top ten results of each analysis were selected for plotting. GO analysis encompasses gene function across cellular component (CC), biological process (BP), and molecular function (MF) terms. KEGG, established in 1995 by the Kanehisa Laboratory at the Center for Bioinformatics, Kyoto University, Japan, serves as a database resource for comprehending advanced functional and biological systems, particularly those derived from large molecular datasets generated by genome sequencing and other high-throughput experimental techniques. Finally, the gene enrichment map was generated using R version 4.02 software.

### Identification of candidate genes

Human placental expression profiles, PhyloP scores, and Residual Variance Intolerance Score percentiles of the genes were integrated to screen candidate genes from the VOUS region. PhyloP scores were obtained using the UCSC genome browser (https://genome.ucsc.edu/), and genes with scores ≥ 0.4 were considered conserved. Gene expression profiles in the human placenta were retrieved from the Expression Atlas (https://www.ebi.ac.uk/). RVIS scores, downloaded from the RVIS website (http://genic-intolerance.org/), were filtered to include scores ≤ 25th percentile for identifying candidate genes.

## Results

### Characteristics of subjects

All 189 POC samples (comprising 244 experimental results) were successfully detected. Chromosomal abnormalities were identified in 121 POC samples (with 176 results from among these samples), while no abnormalities were observed in 68 POC samples, resulting in an overall abnormal detection rate of 64.02% (121/189). The average gestational duration of abortion was 10.5 ± 3.6 weeks (ranging from 4 to 26.6 weeks), with pregnant women having an average age of 30.3 ± 4.4 years (ranging from 21 to 42 years) and undergoing an average of 1.6 ± 0.8 abortions (ranging from 1 to 6 times) (see Table 1). Interestingly, the study indicated the frequency of CNV abnormalities in the early abortion group was significantly higher than that in the late abortion group(*P* < 0.05)(see Table 1).

**Table 1:** Age, gestational age and number of abortions in 189 cases of spontaneous abortion

Factor(mean ± SD)		Maternal age^a^ (30.3 ± 4.4)		Gestational weeks^b^ (10.5 ± 3.6)		Number of abortions^c^(1.6 ± 0.8)	
Group		< 35	≥ 35	< 12 w	≥ 12 w	< 2	≥ 2
Number of cases		159	30	140	49	111	78
Normal	Total	59	9	44	24	38	30
	Proportion %	37.11%	30.00%	31.43%	48.98%	34.23%	38.46%
Abnormalities	Numerical chromosome abnormalities	41	14	43	12	36	19
	CNVs	42	4	35	11	25	21
	Complex abnormalitie	17	3	18	2	12	8
	Total	100	21	96	25	73	48
	Detection rate %	62.89%	70.00%	68.57%	51.02%	65.77%	61.54%
χ 2		0.28788		4.1219		0.19556	
P-value		0.592		0.042		0.658	

* Both numerical abnormalities and CNVs were detected. w, weeks

a. Maternal age: < 35 years old was the appropriate age group, and ≥ 35 years old was the elderly parturient women group

b. Gestational weeks: < 12 weeks for early abortion, ≥ 12 weeks for late abortion

c. Number of abortions: < 2 times were sporadic abortion, ≥ 2 times were recurrent abortion

### Results of numerical chromosome abnormalities and CNVs

**Fig. 1:**
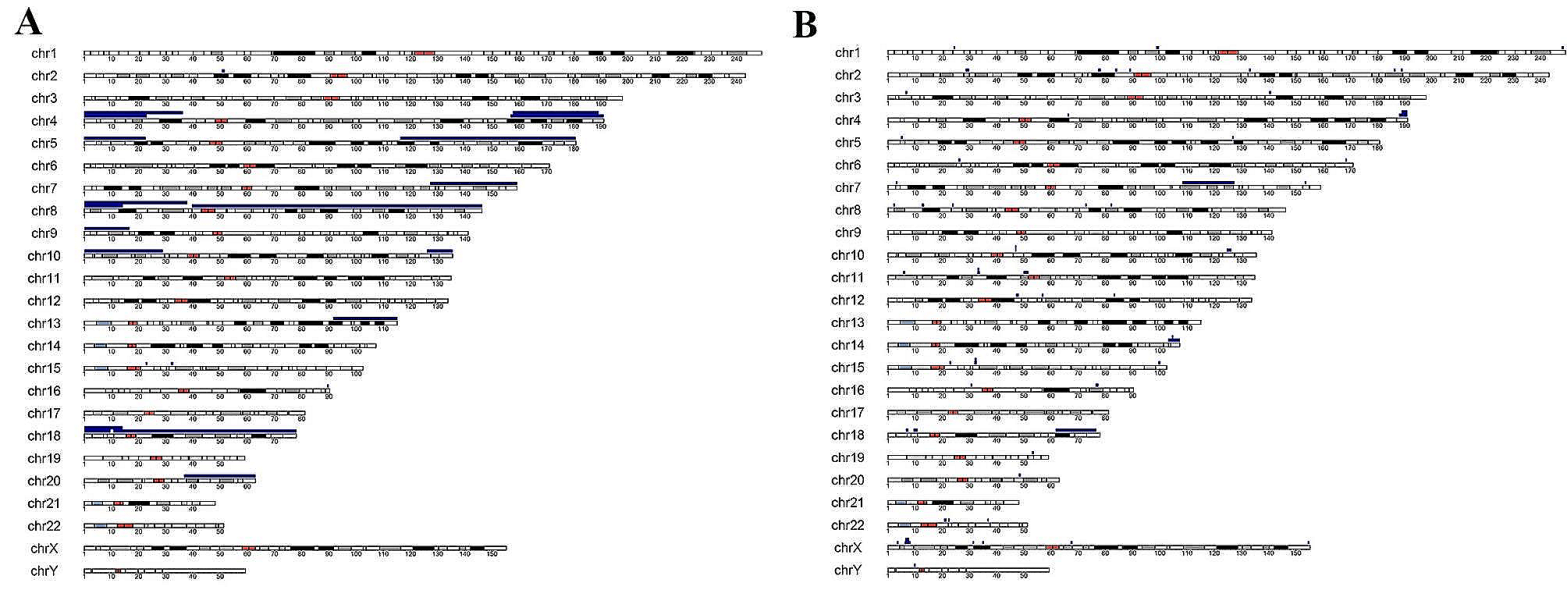
The distribution of pCNVs(**A**) and VOUS CNVs(**B**) on chromosomes

**Fig. 2:**
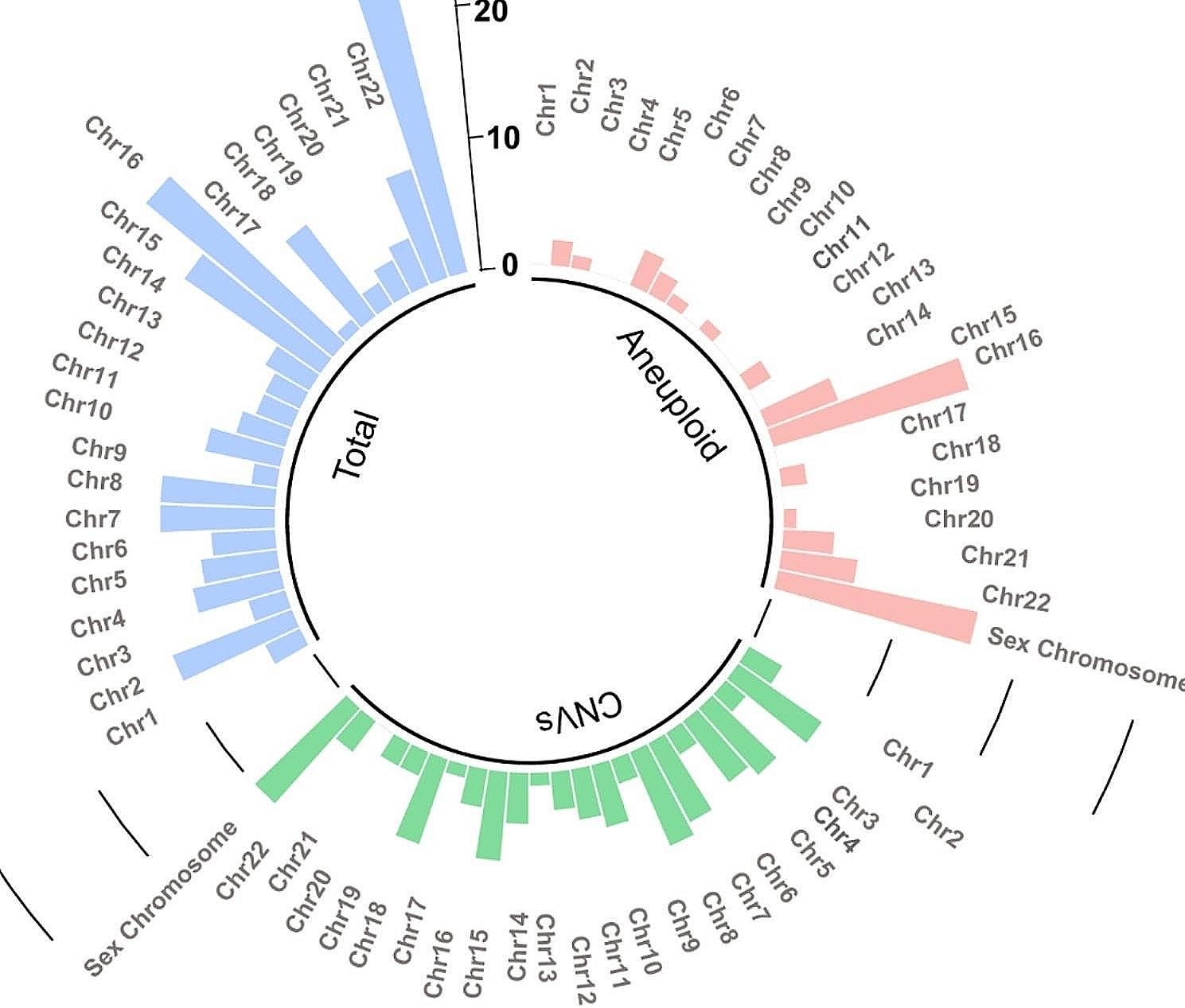
The distribution of aneuploid numerical chromosome abnormalities and CNVs on chromosomes

Among the 121 POC samples, a total of 176 abnormal results (72.13% of 244) were detected, comprising 59 cases (33.52% of 176) of numerical chromosome abnormalities, 73 cases (41.48% of 176) of CNVs, and 44 cases (25.00% of 176) of complex abnormalities where both numerical abnormalities and CNVs were detected (see Tables 2 and 3). Aneuploidy was the most common abnormality among numerical chromosome abnormalities, predominantly involving sex chromosomes and chromosome 16, followed by sex chromosomes in the present POC samples study. Among the CNVs observed, there were 71 duplications and 25 deletions, including 22 pathogenic CNVs (including likely pathogenic CNVs), 66 variants of uncertain significance (VOUS), and 8 likely benign variations. CNVs were detected in all chromosomes except for chromosome 21, with chromosomes X, 8, and 2 being the most frequently affected (see Fig. 1 Fig. 2). Among these cases, Xp22 microduplication (3/71) and 4q3 deletion (3/25) were found (see Supplementary Table 3).

**Table 2:** A total of 244 results in 189 cases

Results		Total	Detection rate
Numerical chromosome		59	33.52%
abnormalities			
	Autosomal trisomy	31	52.54%
	Autosomal monosomy	1	1.69%
	Sex chromosome trisomy	1	1.69%
	Sex chromosome monosomy	12	20.34%
	Euploid	9	15.25%
	Mosaic	5	8.47%
CNVs		73	41.48%
	pCNVs	20	27.39%
	Likely pathogenic CNVs	2	2.74%
	VOUS	46	63.01%
	Likely benign	6	8.22%
Complex abnormalities^*^		44	25.00%
Abnormality		176	72.13%
Normal		68	27.87%

* Both numerical abnormalities and CNVs were detected. CNVs, copy number variations. VOUS, variant of uncertain significance. pCNVs, pathogenic CNVs

**Table 3:** 20 cases of complex abnormalities

Cases	Results	CNVs size	Pathogenic type
Case5	trisomy 22	/	*P*
	seq[hg19]dup(2)(q32.1)chr2:g.188840000_189220000dup	0.38 Mb	VOUS
Case11	trisomy 8	/	*P*
	seq[hg19]dup(Y)(p11.2)chrY: g.9760000_10080000dup	0.32 Mb	VOUS
Case15	trisomy 22	/	*P*
	seq[hg19]dup(11)(p11.12)chr11:g.49980000_51580000dup	1.60 Mb	VOUS
	seq[hg19]dup(7)(q11.1q11.21)chr7:g.61500000_62640000dup	1.14 Mb	LB
Case30	trisomy 16	/	*P*
	seq[hg19]dup(5)(q23.2)chr5:g.126720000_127040000dup	0.32 Mb	VOUS
Case44	48,XN,+13,+22[65%]/49,XN,+13,+16,+22[35%]	/	*P*
	seq[hg19]del(15)(q11.2)chr15:g.22740000_23100000del	0.36 Mb	*P*
	seq[hg19]dup(2)(q32.1)chr2:g.186260000_186600000dup	0.34 Mb	VOUS
Case48	trisomy 15	/	*P*
	seq[hg19]dup(2)(p12)(mos)chr2:g.77360000_78200000dup	0.84 Mb	VOUS
Case50	trisomy 15	/	*P*
	seq[hg19]dup(X)(p21.1)chrX: g.34800000_35160000dup	0.36 Mb	VOUS
Case52	47, XXY	/	*P*
	seq[hg19]dup(18)(p11.31p11.23)chr18:g.7080000_7440000dup	0.36 Mb	VOUS
Case54	trisomy 16	/	*P*
	seq[hg19]dup(1)(p36.11)chr1:g.24260000_24580000dup	0.32 Mb	VOUS
Case58	trisomy 16	/	*P*
	seq[hg19]dup(3)(p26.1)chr3:g.6500000_7000000dup	0.50 Mb	VOUS
Case61	45,X[50%]/46,XY, del(Y)(p11.31-p11.2)[50%]	/	*P*
	seq[hg19]dup(15)(q26.3)chr15:g.99580000_100120000dup	0.54 Mb	VOUS
Case68	trisomy 13	/	*P*
	seq[hg19]dup(22)(q11.22)chr22:g.22260000_22580000dup	0.32 Mb	VOUS
Case74	X monosomy	/	*P*
	seq[hg19]dup(15)(q13.3) chr15:g.31880000_32520000dup	0.64 Mb	VOUS
Case76	trisomy 16	/	*P*
	seq[hg19]dup(12)(q13.3)chr12:g.56660000_57120000dup	0.46 Mb	VOUS
	seq[hg19]dup(X)(q12)chrX: g.67340000_67720000dup	0.38 Mb	VOUS
Case88	X monosomy	/	*P*
	seq[hg19]dup(8)(p21.2)chr8:g.23760000_24100000dup	0.34 Mb	VOUS
Case100	trisomy 7	/	*P*
	47, XYY	/	*P*
	seq[hg19]dup(15)(q11.2q11.2)chr15:g.22740000_23100000dup	0.36 Mb	VOUS
Case107	trisomy 6	/	*P*
	seq[hg19]del(9)(p23p23)chr9:g.11780000_12140000del	0.36 Mb	LB
Case122	trisomy 16	/	*P*
	seq[hg19]dup(2)(p11.2p11.2)chr2:g.88840000_89280000dup	0.44 Mb	VOUS
Case144	trisomy 22	/	*P*
	seq[hg19]dup(20)(q13.13q13.13)chr20:g.48240000_48760000dup	0.52 Mb	VOUS
Case150	trisomy 16	/	*P*
	seq[hg19]dup(15)(q13.3q13.3)chr15:g.32020000_32460000dup	0.44 Mb	VOUS

pCNVs: pathogenic CNVs; VOUS: variant of uncertain significance

### Functional enrichment analysis of pCNVs and VOUS

**Fig. 3:**
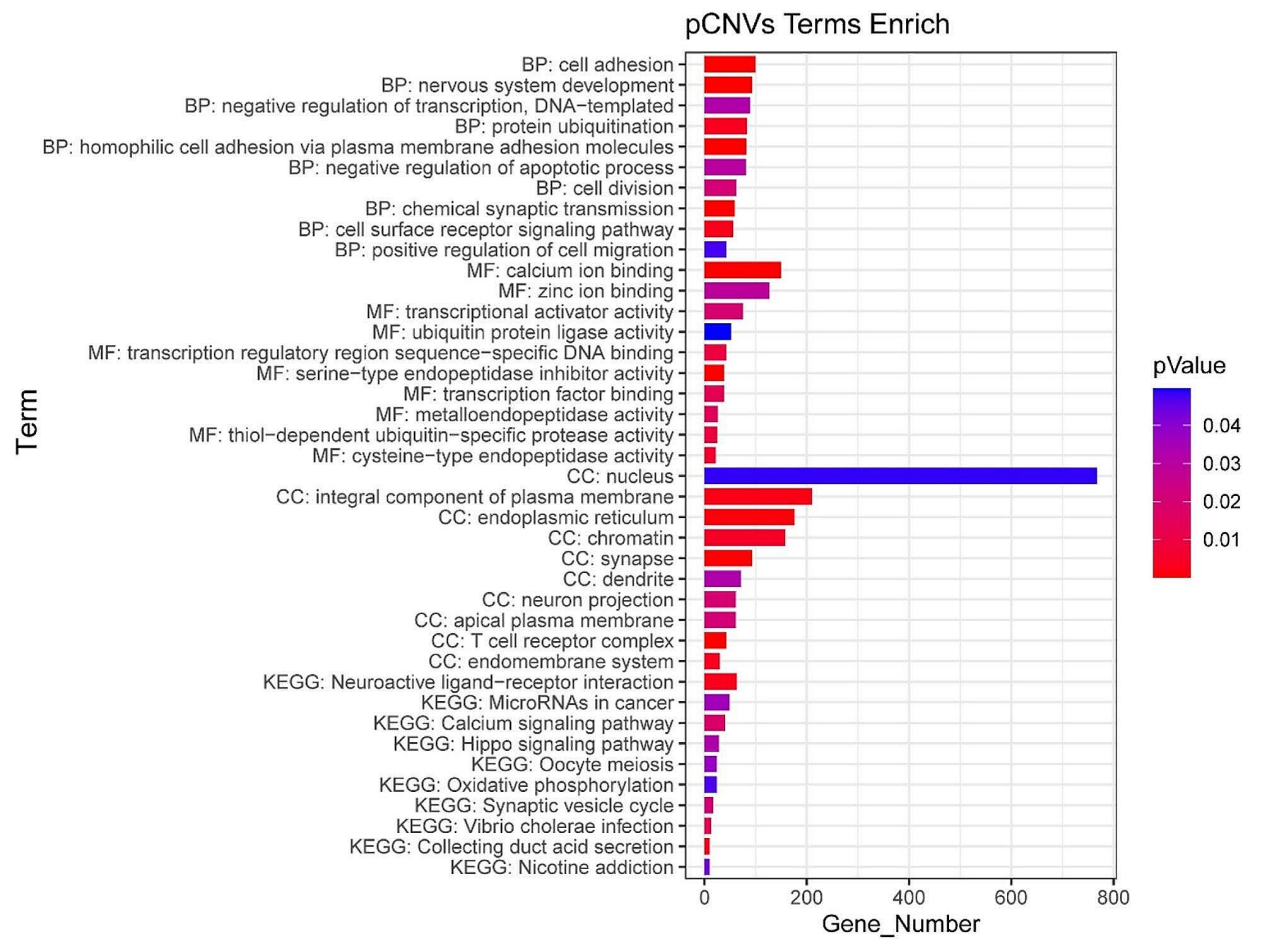
The top 10 pCNVs enrichment results (*P* < 0.05) of analysis using the Gene Ontology and Kyoto Encyclopedia of Genes and Genomes. MF, Molecular Function; CC, Cellular Component; BP, Biological Process

**Fig. 4:**
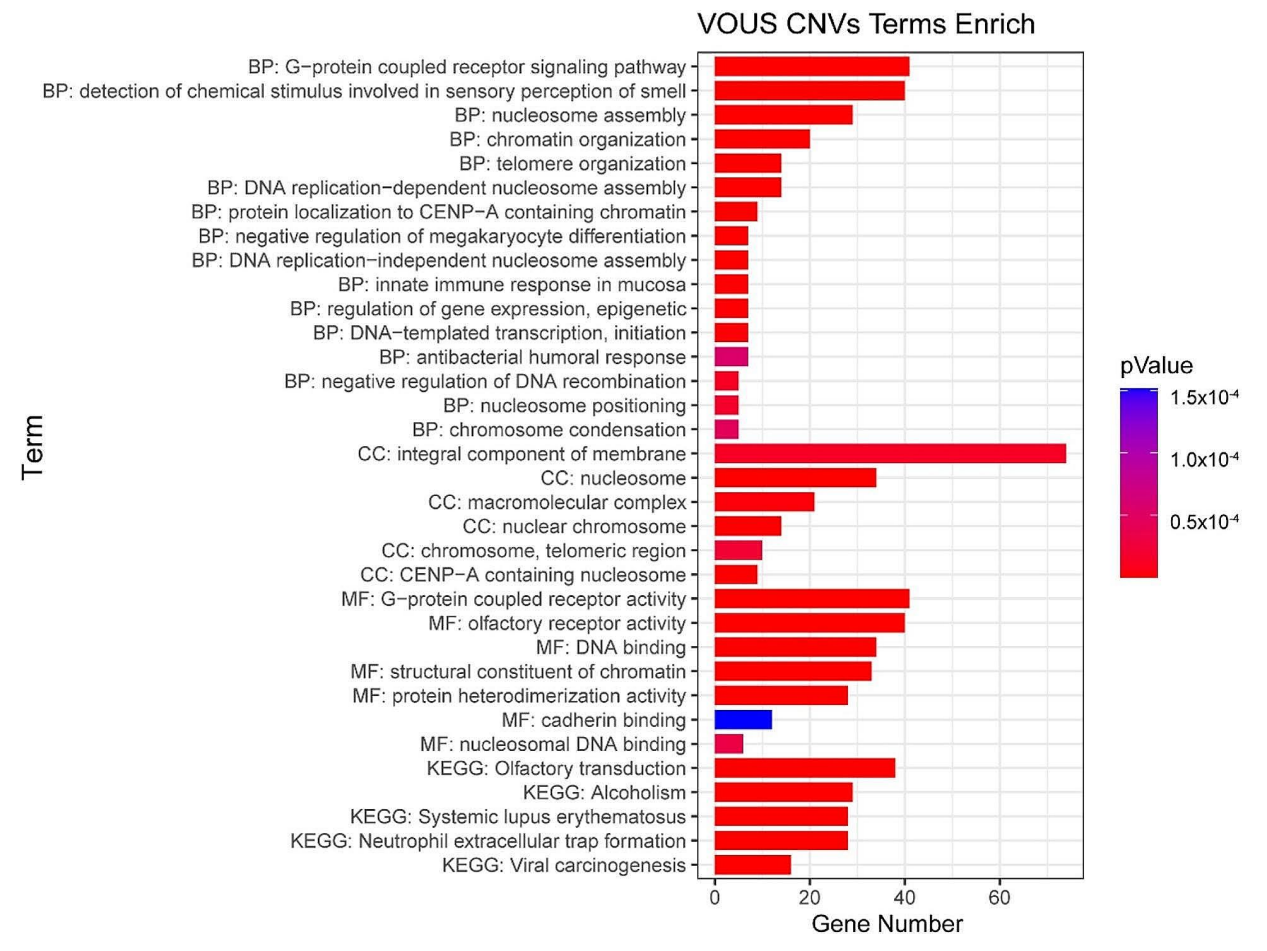
The VOUS CNVs enriched results (*P* < 0.05) of analysis using the Gene Ontology and Kyoto Encyclopedia of Genes and Genomes. MF, Molecular Function; CC, Cellular Component; BP, Biological Process

We conducted gene enrichment analysis of genes from pathogenic CNVs (including likely pathogenic CNVs) and VOUS regions. The analysis revealed 4277 genes in pathogenic CNVs and 188 genes in VOUS CNVs. GO analysis indicated significant enrichment of 205 different functions (*P* < 0.05) among the 4277 genes from pathogenic CNVs (see Fig. 3 and Supplementary Table 1), and 29 different functions (*P* < 0.05) among the 188 genes from VOUS CNVs, with the most significant functions being “homophilic cell adhesion via plasma membrane adhesion molecules” (*P* = 1.35 × 10^-29) and “structural constituent of chromatin” (*P* = 3.76 × 10^-42), respectively (see Fig. 4 and Supplementary Table 2). KEGG analysis of pathogenic CNVs identified “Neuroactive ligand-receptor interaction” (*P* = 0.002) as the most commonly enriched signaling pathway among the 11 pathways identified (*P* < 0.05), while KEGG analysis of VOUS CNVs revealed “Systemic lupus erythematosus” (*P* = 9.42 × 10^-26) as the most enriched among the 5 pathways identified (*P* < 0.05).

The GO and KEGG analysis results mentioned above indicated the enrichment of several biological processes, including nervous system development, transmembrane transport, cell adhesion, and structural constituent of chromatin.

### Identification of candidate genes from VOUS CNVs

We used human placental expression profiles, PhyloP scores, and RVIS percentiles of genes encompassed in detected CNVs to identify potential candidate genes. PhyloP scores reflect the evolutionary conservation of genes, with higher scores indicating greater conservation. RVIS percentiles assess the susceptibility of genes to genetic variations, with values below 25% indicating intolerance to mutations, implying a higher probability that disruption of the gene is pathogenic. After excluding cases with complex abnormalities, we further analyzed a total of 31 CNVs in 24 cases. These CNVs encompassed 188 genes. Ultimately, we identified 14 genes with PhyloP scores greater than 0.4 and RVIS percentiles below 25%, each found in four cases (*LZTR1, TSHZ1, AMIGO2, H1-4, H2BC4, H2AC7, H3C8, H4C3, H3C6, PHKG2, PRR14, RNF40, SRCAP, ZNF629*), with the *LZTR1* gene possibly associated with SA (see Table 4).

**Table 4:** 14 candidate genes were screened out in 4 cases

Case	Maternal age	Gestational weeks	Number of abortions	Gene	Cytoband	Type	PhyloP	human placental expression profile	RVIS_ExAC %
Case1	26	12^+ 1^	2	LZTR1	22q11.21	del	0.42968	16 TPM	1.37%
Case2	28	11^+ 5^	3	TSHZ1	18q22.3	del	0.512115	64 TPM	4.18%
Case3	30	7^+ 0^	2	AMIGO2	12q13.11	dup	1.2425	36 TPM	13.03%
Case4	28	10^+ 6^	1	H1-4	6p22.2	dup	4.28406	265 TPM	22.11%
				H2BC4	6p22.2	dup	3.37532	116 TPM	23.36%
				H2AC7	6p22.2	dup	4.27069	28 TPM	23.42%
				H3C8	6p22.2	dup	4.26379	423 TPM	23.61%
				H4C3	6p22.2	dup	4.42397	40 TPM	24.55%
				H3C6	6p22.2	dup	4.28406	265 TPM	22.11%
				PHKG2	16p11.2	dup	0.551319	12 TPM	11.38%
				PRR14	16p11.2	dup	0.692316	29 TPM	14.18%
				RNF40	16p11.2	dup	0.654751	37 TPM	3.95%
				SRCAP	16p11.2	dup	0.65965	6 TPM	0.23%
				ZNF629	16p11.2	dup	0.441559	14 TPM	23.18%

## Discussion

Chromosomal abnormalities in embryos are well recognized as being associated with the risk of spontaneous abortion. In the past, molecular diagnosis of POCs was conducted using chromosome karyotype analysis, but its lengthy experimental period and low resolution resulted in numerous misdiagnoses. With the rapid advancement of next-generation sequencing technology, CNV-seq has become widely adopted in clinical practice, significantly improving the rate of abnormality detection. According to the guidelines of the American College of Medical Genetics and Genomics (ACMG) [26], results are interpreted as pathogenic CNVs, likely pathogenic CNVs, variants of uncertain significance CNVs, likely benign, and benign CNVs. VOUS CNVs have been identified in many embryos or fetuses with developmental abnormalities or abortion. The impact of these VOUS CNVs on the normal development of embryos or fetuses remains unknown and requires further investigation through extensive clinical cases and studies. Some genes, such as *THSD1*, have already been associated with embryo development; mutations in these genes can lead to improper blood vessel formation, resulting in embryo death [27]. In this study, we aimed to investigate whether any of the 188 genes were related to embryo and fetus development or spontaneous abortion. We analyzed these genes and ultimately identified 14 candidate genes in 4 cases. Among the 14 genes screened, only the chromosome regions involved in *LZTR1* and *TSHZ1* exhibited copy number deletion, while the others showed duplications.

*LZTR1* serves as a substrate adaptor for the cullin 3 (CUL3) ubiquitin ligase complex and acts as a negative regulator of the Receptor Tyrosine Kinase/Ras GTPase/MAP kinase (RTK/Ras/MAPK) signaling pathway activation [28]. Previous studies have implicated *LZTR1* mutations in conditions such as glioblastoma (GBM), schwannomatosis (SWNMT), and Noonan syndrome (NS) [29–31]. We identified a 0.74 Mb deletion from case 1 at chromosome 22q11.2 region, which contains the *LZTR1* gene located in the central region of 22q11.2 deletion syndrome (Velocardiofacial/DiGeorge syndrome) manifested as severe congenital heart disease, cleft palate and other specialized facial features, and severe immunodeficiency. Previous studies have reported two cases with similar microdeletions to the present case with clinical phenotype of developmental delay, language developmental disorders, mental retardation, and peculiar facial features [32, 33]. We hypothesize that the microdeletion of the central chromosome 22q11.2 region may lead to severe cardiovascular problems and immune deficiencies that result in embryo termination. The RTK/Ras/MAPK pathway plays a significant role in regulating cell proliferation and survival, apoptosis, differentiation, and nervous system function. Inactivation of *LZTR1* leads to decreased ubiquitination, resulting in the overactivation of the RTK/Ras/MAPK signaling pathway [34]. The overactivation of Ras/MAPK pathways leads to increased cell division and proliferation, resulting in the excessive accumulation of reactive oxygen species (ROS), ultimately activating the apoptotic pathway. During embryonic cell development, metabolic reactions occur, leading to the production of aging mitochondria. When this pathway becomes dysregulated, excessive accumulation of aging mitochondria and ROS ensues, leading to cellular toxicity and enhanced oxidative stress, ultimately resulting in cell apoptosis. Numerous studies have suggested that oxidative stress plays a crucial role in early pregnancy loss [35]. ROS is closely linked to various aspects of the female reproductive process, particularly in the ovaries and embryos [36]. ROS exerts biological effects on various reproductive processes. It can be inferred that the loss of *LZTR1* leads to the inability to negatively regulate the signaling pathway, resulting in excessive pathway activation and ROS accumulation. Oxidative stress disrupts placental trophoblast function [37], and also plays a role in regulating the reproductive process signaling pathway, altering the uterine immune system and leading to embryo failure [38].

The histone family comprises histones H1, H2A, H2B, H3, and H4, representing evolutionarily conserved protein families. This family is associated with developmental disorders and various neoplasms [39–41]. Histones play crucial roles in transcriptional regulation and DNA replication [42, 43]. Mutations in *H1-4* and *H4C3* are implicated in syndromes characterized by intellectual disability. *H4C3* is particularly crucial in embryonic development [39], with mutations in this gene in zebrafish models resulting in severe embryonic developmental defects. *TSHZ1* is linked to congenital aural atresia and anosmia; Tshz1-/- leads to neonatal lethality in mouse experiments [44]. Among other genes, *AMIGO2* and *PHKG2* are associated with gastric adenocarcinoma and glycogen storage disease, respectively [45, 46]. *SRCAP* encodes an ATPase and is linked to developmental delays and Floating-Harbor syndrome (FHS) when this gene loses function [47], FHS is a rare genetic disease typically manifesting in early childhood, characterized by short stature and facial dysmorphism. While this study suggests that *PRR14*, *RNF40*, and *ZNF629* genes may be associated with embryonic development, neonatal lethality, or abortion, the sample size is not sufficient. Otherwise, we did not investigate whether these CNVs were inherited or de novo, and follow-up studies remained to be continued. Thus, more CNV-seq results of POCs are required to identify genes associated with SA. Some genes have demonstrated roles in embryonic development in animal studies; however, further basic experiments are necessary to validate their functions and mechanisms in vivo.

## Conclusion

In this study, we sequenced the tissue samples from 189 cases of spontaneous abortion and integrated various gene scores to screen for genes involved in the VOUS CNVs region detected in spontaneous abortion samples. Among 188 genes analyzed, we identified 14 potential developmental genes, with most being associated with neurodevelopment and signaling pathway regulation. Our findings suggest that *LZTR1, TSHZ1* and *H4C3* genes are likely linked to embryonic development, offering new insights into the pathogenesis of SA.

### Electronic supplementary material

Below is the link to the electronic supplementary material.

Supplementary Material 1

Supplementary Material 2

Supplementary Material 3

## Data Availability

No datasets were generated or analysed during the current study.
